# Common Food-Wrap Film as a Cost-Effective and Readily Available Alternative to Thermoplastic Polyurethane (TPU) Membranes for Microfluidic On-Chip Valves and Pumps

**DOI:** 10.3390/mi16060657

**Published:** 2025-05-30

**Authors:** Huu Anh Minh Nguyen, Mark Volosov, Jessica Maffei, Dae Jung Martins Cruz, Roman Voronov

**Affiliations:** 1 Otto H. York Department of Chemical and Materials Engineering, Newark College of Engineering, New Jersey Institute of Technology, University Heights, Newark, NJ 07102, USA; hn254@njit.edu; 2 Helen and John C. Hartmann Department of Electrical and Computer Engineering, Newark College of Engineering, New Jersey Institute of Technology, University Heights, Newark, NJ 07102, USA; mv7775050@gmail.com (M.V.); dm95@njit.edu (D.J.M.C.); 3 Department of Biomedical Engineering, Newark College of Engineering, New Jersey Institute of Technology, University Heights, Newark, NJ 07102, USA; jfm23@njit.edu

**Keywords:** microfluidics, rapid prototyping, laser cutting, on-chip valves, integrated microvalves and micropumps, membranes, food-wrap film

## Abstract

Microfluidic devices rely on precise fluid control to enable complex operations in diagnostics, chemical synthesis, and biological research. Central to this control are microvalves, which regulate on-chip flow but require flexible membranes for active operation. While the laser cutting of thermoplastics offers a fast, automated method for fabricating rigid microfluidic components, integrating flexible elements like valves and pumps remains a key challenge. Thermoplastic polyurethane (TPU) membranes have been adopted to address this need but are costly and difficult to procure reliably. In this study, we present commercial food-wrap film (FWF) as a low-cost, widely available alternative membrane material. We demonstrate FWF’s compatibility with laser-cut thermoplastic microfluidic devices by successfully fabricating Quake-style valves and peristaltic pumps. FWF valves maintained reliable sealing at 40 psi, maintained stable flow rates of ~1.33 μL/min during peristaltic operation, and sustained over one million continuous actuation cycles without performance degradation. Burst pressure testing confirmed robustness up to 60 psi. Additionally, FWF’s thermal resistance up to 140 °C enabled effective thermal bonding with PMMA layers, simplifying device assembly. These results establish FWF as a viable substitute for TPU membranes, offering an accessible and scalable solution for microfluidic device fabrication, particularly in resource-limited settings where TPU availability is constrained.

## 1. Introduction

Microfluidic devices provide precise control of fluids at microscales, facilitating advances in diagnostics, chemical synthesis, and biological research. Their versatility relies critically on microvalves that enable programmable fluid routing and isolation within microfluidic networks, essential for automated workflows.

Over the past two decades, diverse valve designs have emerged, including passive Tesla valves [1], cantilever-based valves [2,3], diaphragm valves [4], and pneumatically actuated elastomeric valves inspired by the original Quake design [5]. Despite their variations, active microvalves consistently depend on flexible membranes for dynamic flow control.

Traditionally, microfluidic devices with flexible valves were fabricated using photolithography and polydimethylsiloxane (PDMS) soft lithography. While effective, these methods are slow, labor-intensive, costly due to mask production, prone to user error, and difficult to scale and produce devices with limited chemical resistance and short operational lifespans [6].

To overcome these limitations, the laser cutting of thermoplastics such as polymethyl methacrylate (PMMA), polycarbonate (PC), and cyclic olefin copolymer (COC) has gained popularity [7]. Laser cutting enables the rapid, automated, and scalable fabrication of rigid microfluidic components, enhancing durability and chemical resistance compared to PDMS-based devices. However, integrating flexible, membrane-based valves within these rigid thermoplastic systems remains challenging, primarily because the flexible membrane must be precisely sandwiched between rigid layers and then securely bonded, typically requiring specialized bonding techniques to ensure structural integrity and leak-free performance. Thermoplastic polyurethane (TPU) membranes, specifically the PT9200US NAT 1.0 mil film from Covestro LLC, have been employed to address this, owing to their excellent mechanical properties and chemical compatibility [8,9,10,11,12,13,14]. Yet, TPU is expensive and difficult to procure and often requires custom orders, introducing significant delays and supply chain issues.

Motivated by these challenges, we propose an alternative: readily available, inexpensive (~USD 1/m^2^), commercial food-wrap film (FWF) based on polyvinylidene chloride (PVDC). Due to its accessibility and affordability, FWF is particularly attractive for resource-limited settings and rapid, low-cost prototyping applications. While a comprehensive comparison of all alternative materials would be valuable, to our knowledge, no alternative materials have been reported in the literature for flexible membranes used in laser-cut microfluidic valve assemblies. Given TPU’s procurement difficulties and the absence of documented alternatives, FWF represents the first reported substitute with similar properties for this specific application.

Consequently, FWF is exceptionally well suited for developing disposable, single-use microfluidic devices, particularly for applications such as point-of-care diagnostics, where affordability and the prevention of cross-contamination are critical design considerations. The demonstrated ease of fabrication and integration of FWF into functional on-chip valves and pumps further supports its utility in these scenarios. The incorporation of such an accessible membrane material could also accelerate the design and iteration of more complex microfluidic systems requiring sophisticated active flow control, enabling a shift from basic channel networks to integrated ‘lab-on-a-chip’ platforms.

The PVDC-based FWF employed in this study offers inherent advantages beyond its primary benefits of low cost and wide availability. PVDC is recognized for its excellent barrier properties against oxygen and moisture, beneficial for microfluidic applications requiring controlled atmospheric conditions or preventing reagent evaporation. Its designated use in food-contact baking applications suggests chemical inertness and material safety advantages for certain biological or chemical assays. Furthermore, its notable thermal resistance up to 140 °C facilitates robust thermal fusion bonding with PMMA layers and signifies stability for on-chip applications involving temperature cycling or localized heating.

In this study, we systematically evaluate FWF as a membrane for fabricating elastomeric components in Quake-style valves and peristaltic pumps, focusing on mechanical durability, integration potential, and operational performance. While the present study addresses the mechanical functionality of FWF as a direct replacement for TPU in valves and pumps, comprehensive investigations into its chemical compatibility and biocompatibility for diverse applications constitute important avenues for future research.

Thus, to the best of our knowledge, this study provides the first alternative to TPU membranes for laser-cut microfluidic devices. Demonstrating comparable functionality and robustness, FWF represents a practical solution for overcoming logistical and financial constraints inherent in membrane-based microfluidic technologies, significantly benefiting resource-limited environments and disposable, single-use applications.

## 2. Materials and Methods

### 2.1. Microfluidic Device Layout

**Figure 1:**
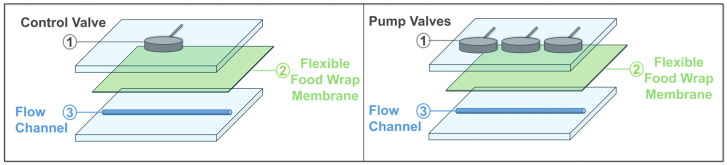
The layouts of microfluidic devices with FWF membrane for single-valve and peristaltic pump configurations. LEFT: The schematic on the left shows a single-valve configuration used for controlling fluid flow through a straight channel. RIGHT: The schematic on the right illustrates a peristaltic pump setup with three sequentially placed valves, enabling directional fluid movement through the channel. Each configuration consists of three layers: (1) the top layer containing the control or pump valves, (2) a middle layer with the flexible food wrap membrane (FWF), and (3) the bottom layer with the flow channel.

The microfluidic device used for testing the FWF-based on-chip valve is illustrated in Figure 1. For simplicity, a straight channel configuration with a single valve was chosen (or three valves in the case of the peristaltic pump). The device was composed of three main layers: (1) a top control layer containing the control valve, (2) a middle flexible layer made of FWF acting as the membrane, and (3) a bottom layer containing the straight flow channel.

**Figure 2:**
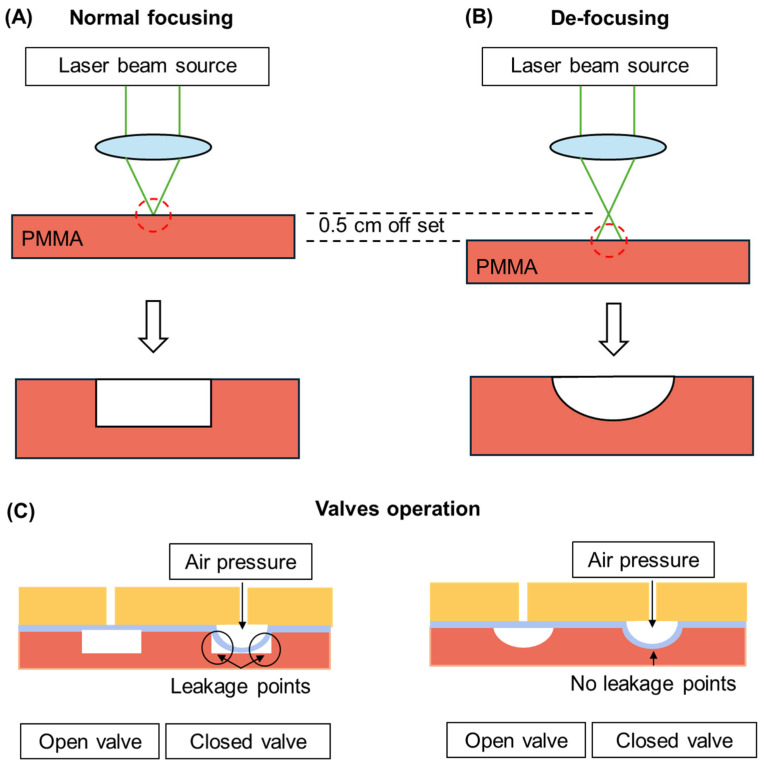
Illustrations demonstrating the laser engraving process of rounded-profile channels and the valves’ operation, showing why they are preferred over rectangular profile ones for the fabrication of Quake-style microfluidic valves. Defocusing in the laser engraver is achieved by moving the platform by an offset distance (**A**,**B**). Regarding differences in valves’ operation, an inflated membrane is unable to perfectly seal square corners of a square channel (**C-left**), resulting in leakage. In contrast, the rounded channel in (**C-right**) results in a perfect seal.

The flow channels were designed with a semi-circular profile to enhance valve sealing performance and prevent leakage when the valve was actuated (see Figure 2). These rounded profiles were achieved through the deliberate defocusing of the laser during fabrication (discussed in detail in Section 2.3). The control channels, in contrast, had a square profile and were in the top layer above the FWF membrane. This configuration formed a Quake-style valve [5], where the combination of a semi-circular flow channel and a square control channel ensured a tight seal during operation.

The device was fabricated with flow channel dimensions of 0.940 mm in width and 27.2 mm in length. The channel depth was measured to be approximately 0.4 mm. The microvalves were designed with diameters approximately 9 times the flow channel width to ensure a sufficient contact area for effective sealing. For the flow and valve fatigue experiments, the control channels had dimensions of 0.650 mm for width, 3.69 mm for length, and 0.4 mm for depth, while the corresponding valve diameter was 9.16 mm. For the peristaltic pump experiments, a set of three microvalves was used, each with a diameter of 5.12 mm and depth of 0.4 mm.

These carefully designed dimensions and configurations provided an effective platform for evaluating the performance and reliability of the FWF membrane in on-chip microfluidic applications.

### 2.2. Materials

Polymethyl methacrylate (PMMA) was used in the form of 3 mm casted clear acrylic sheets sourced from Makerstock (Old Forge, PA, USA). High-purity isopropyl alcohol (IPA, >99%, Sigma Aldrich, St. Louis, MO, USA) and chloroform (>99%, ThermoFisher, Waltham, MA, USA) were employed to clean and treat the surfaces of the laser-cut acrylic slabs, respectively.

A FWF (Asahi Saran, ASIN- B0CBB9CZ82, Amazon, Asahi Kasei, Tokyo, Japan) was used as the flexible membrane for the on-chip microvalves and micropumps. This PVDC-based FWF is designed for baking applications and can withstand high temperatures up to 140 °C. The high thermal resistance of this material was a crucial factor in its selection, as it ensures that the membrane remains intact during the thermal fusion bonding process with the PMMA slabs. Non-baking FWFs were not used due to their tendency to melt under these high-temperature conditions.

### 2.3. Laser Cutting

Microfluidic channels were fabricated in the PMMA sheets using a commercial CO_2_ laser-cutting machine (Speedy 100 model, New Bern Trotec, NC, USA) at the New Jersey Institute of Technology (NJIT)’s Makerspace. The laser-cutter was equipped with a 24 × 12-inch X-Y platform, an adjustable *Z*-axis, and a 1.5-inch focus lens. The designs for the microfluidic geometries were created using CorelDRAW Graphics Suite X8 (version 18.0) and then transferred to Trotec’s JobControl software (version 11.4.0, Trotec, New Bern, NC, USA) for laser processing. The optimal laser cutting parameters used in this study were 35% power, 35% speed, a pulse frequency of 1000 Hz, and a single pass.

**Figure 3:**
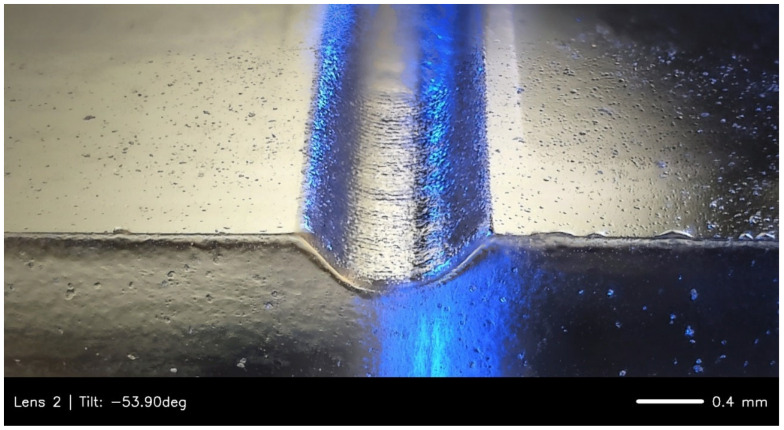
A representative tilted view brightfield microscopy image of a laser-engraved microfluidic channel’s rounded profile. The semi-circular channel profiles were achieved by defocusing the laser during engraving. The channel has been illuminated with blue light to enhance the surface contrast and highlight the curvature and texture of the channel walls.

To ensure a rounded channel profile essential for the leak-free operation of on-chip Quake-style [5] microfluidic valves, the laser beam was deliberately defocused out of the normal focus plane [8]. Figure 3 shows a representative result, which was obtained by placing the PMMA surface at the exact focal plane distance of the laser’s 1.5-inch lens and then applying an offset Z-distance of 0.2 inches (~5 mm) in the software to defocus the beam.

### 2.4. Surface Treatment of the Laser-Engraved PMMA Sheets

Following the creation of the device features, the laser-engraved PMMA sheets were cleaned by submerging and washing them twice with IPA. To smooth out rough edges and eliminate any debris generated by the high-temperature laser cutting, the surfaces were then exposed to chloroform vapor for 4 min. This step was crucial, as the subsequent bonding process required smooth surfaces to ensure a strong and effective seal between the layers.

For the vapor exposure, the PMMA sheets were placed above a container filled with chloroform, ensuring that they did not come into direct contact with the liquid. This procedure was conducted at room temperature inside a fume hood to ensure safety and prevent the inhalation of fumes.

### 2.5. Heat-Press Thermal Bonding

**Figure 4:**
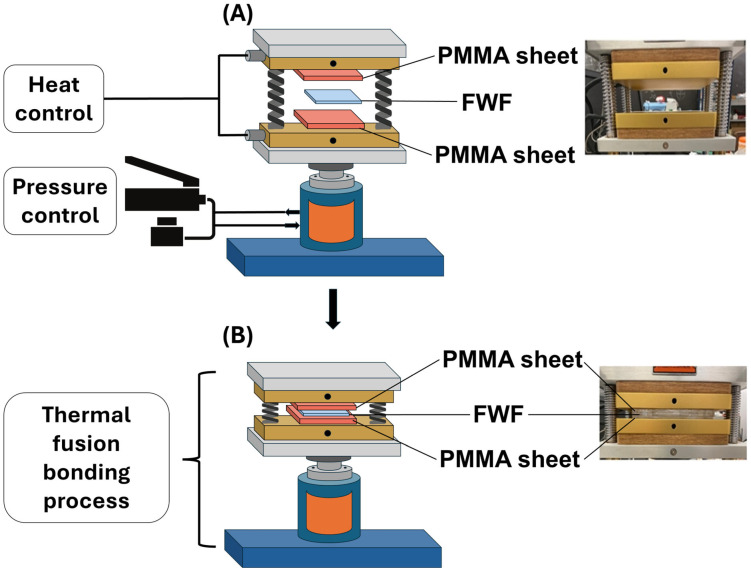
The schematic and setup for the heat-press thermal fusion bonding of the PMMA and FWF membranes, with the figures illustrating the thermal bonding process used to assemble the microfluidic device. The schematic in (**A**) shows the sandwich structure of the PMMA sheets and FWF membrane and the heat press equipment setup before the process. The bottom figures in (**B**) show the thermal bonding step, with the plates being pressed and heated to create the right conditions for temperature and pressure for the PMMA sheets and FWF membrane inside. This process ensures a strong, leak-free bond between the PMMA sheets and the flexible membrane, critical for the functionality of the microfluidic valves and channels.

A heat press equipped with dual (top and bottom) heating plates (dp-hr20t77, Dabpress, Shenzhen, Guangdong, China) was used for the thermal bonding [15] of the PMMA sheets and the FWF membrane. Achieving a high-quality seal required minimizing sample deformation, preventing leakage between layers, and ensuring a strong, irreversible bond through thermal fusion bonding. The overall process is shown in Figure 4.

**Figure 5:**
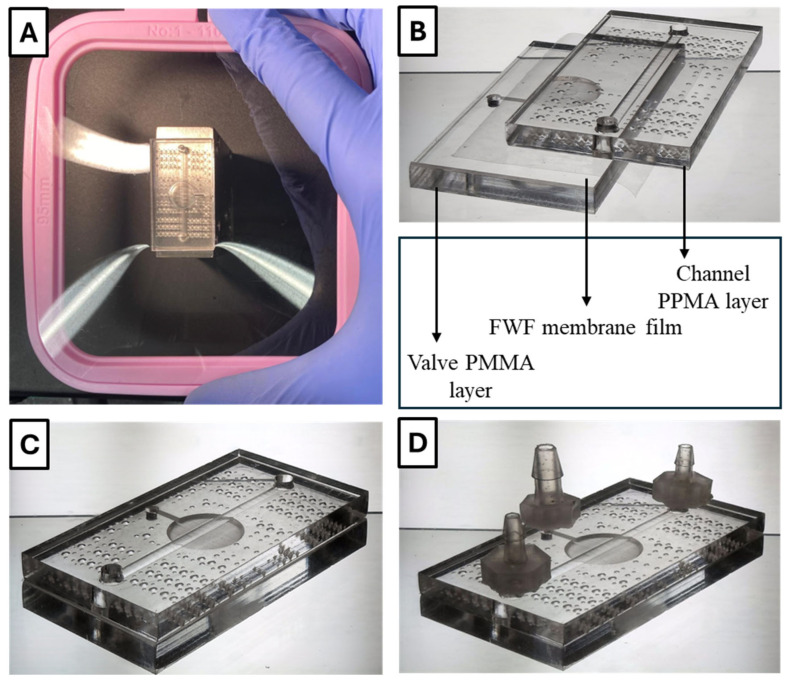
The fabrication and assembly process of the FWF-based microfluidic device. The figure illustrates key steps in the fabrication and assembly of the FWF-based microfluidic device. (**A**) Prior to being cut, the FWF membrane was secured in place using a 110 mm sewing frame to ensure smooth and uniform contact between the PMMA sheets and the membrane. (**B**) A tilted view of the resulting layer assembly “exploded” to show the top PMMA layer with the valve control channel and laser-engraved bubble traps, the FWF membrane in the middle, and the bottom PMMA layer with the fluid channel. (**C**) A titled view of the device whose layers have been aligned and heat-pressed. (**D**) A titled view of the fully assembled device with barbs installed for fluidic connections. This comprehensive assembly process highlights the fabrication techniques used to achieve reliable microfluidic functionality. Panes B-D were assembled into panoramas from separate tiles taken using the Microqubic 3D microscope, and then uneven illumination was corrected in them via Background Subtraction using ImageJ Fiji software version 1.5f [16].

One challenge in the bonding process arises from the gas-impermeability of both PMMA and FWF, which can trap air bubbles at the PMMA/FWF interface, reducing bonding strength. To mitigate this issue, we incorporated laser-engraved air bubble traps in non-critical areas of the PMMA surfaces (see Figure 5C and Supplementary Figure S4). Additionally, the PMMA substrates underwent a 2 min plasma treatment in a plasma cleaner (PDC-001 plasma cleaner, Harrick Plasma, Ithaca, New York, USA) to enhance surface adhesion before bonding. The FWF membrane was then carefully applied and secured using a 110 mm sewing frame (Pllieay embroidery hoops, Pllieay, ASIN: B093GXHSSR, Amazon, Shenzhen Tongzhou Technology Co., Ltd., Shenzhen, China) to maintain a smooth, flat, and uniform contact between the PMMA and FWF layers (see Figure 5A).

Following the surface preparations, the device layers were aligned and sandwiched between two Polytetrafluoroethylene (PTFE) Teflon sheets (0.11 mm thick Teflon sheets, YRYM HT, ASIN: B07H55M1ZR, Amazon, HUNAN SIJIU E-COMMERCE CO., LTD, Changsha, Hunan, China) and two silicone mats (0.33″ thick silicone mats, Shopley, ASIN: B093GXHSSR, Amazon, Seattle, Washington, USA) cut to fit the heating plates. These materials ensured that the chips remained in place and did not deform under the high pressure of the heat press. The aligned “sandwich” assembly (see Figure 5B) was subjected to a series of temperature treatments, 180 °F, 200 °F, 220 °F, and 240 °F, with each applied for 10 min. This was followed by gradual cooling to room temperature (77 °F), which took approximately 40 min to complete the bonding.

### 2.6. Actuation System for Microfluidic Valves and Pumps

To enable effective fluidic and pneumatic connections, barbs were bonded to the top surface of the microfluidic chips using superglue (GH1200, cyanoacrylate glue, KTC Global LLC., Cincinnati, OH, USA) (see Figure 5C,D). These barbs were essential components that served as the world-to-chip interface, allowing for secure attachment to the tubing systems used for liquid and gas input/output operations.

The actuation of the fabricated microvalves and micropumps was controlled using a custom-built valve controller system [17,18,19]. This system employed a Wago controller (PFC100; 2 x ETHERNET; ECO, WAGO GmbH & Co. KG, Minden, Germany) to operate solenoid valves (Festo, Mason, OH, USA), providing precise control over fluid flow and valve actuation. The entire setup was managed through a custom software interface developed in MATLAB R2023b, which automated and synchronized the operations of our microfluidic devices.

### 2.7. Performance Testing of the FWF Membrane Valve

The performance and durability of the FWF membrane valve were tested over a continuous period of 21 days, operating at a frequency of 0.5 Hz. Actuation frequency was limited to 0.5 Hz due to the pneumatic system response time and to match typical biological application requirements. Every 24 h (or 43,200 cycles), the valves were subjected to functional testing to assess their ability to stop fluid flow without any leakage. Direct comparison with TPU membranes under identical experimental conditions was not performed due to procurement difficulties. Performance comparisons were based on literature values from published TPU studies conducted under similar operating conditions. 

A colored liquid, created by mixing water and red food dye, was used to easily visualize the fluid flow within the channels. For the leakage flow rate experiment, the tests were conducted by operating the valve under incrementally increasing actuation pressures in separate tests (0, 10, 20, 30, 40, 50, and 60 psi). During each test, a constant inlet flow pressure of 5 psi was applied for a duration of 1 min. Any fluid that passed through the valve—due to incomplete sealing—was collected at the outlet into a container. The volume of leaked fluid was then measured using a calibrated volumetric pipette to quantify the leakage per condition. Unless otherwise specified, the input of the liquid was carried out by a syringe pump (Pico 11 Elite, Harvard Apparatus Inc., Holliston, MA, USA) at a 0.1 µL/min flow rate, using a plastic 10 mL syringe. For the pressure-based flow rate, a positive pressure system comprising a container, pressure gauges, and a pressure source from the building’s house pressure was used to vary input flow pressure. For varying valve pressure, the custom-built valve controller system was used (see Section 2.6).

The visualization of fluid flow and valve functionality was performed using a Microqubic 3D MRCL700 microscope (CN Tech, Wisbech, UK), with a transmitted (brightfield) light source and the stock “medium zoom” #2 lens from the manufacturer. Image correction was carried out using ImageJ 1.5f (Fiji) software [16].

All flow rates were measured as a function of volume and time as follows:

$$Q=\frac{V}{t}$$

where Q is the flow rate (µL/min), *V* is volume (µL), and t is time (minutes). In all experiments where the flow rate was involved, the output liquid was collected in a container, in a predetermined time, and measured using pipettes, and then the information of volume and time was used to calculate flow rates.

The peristaltic pump actuation frequency was calculated as $f=\frac{1}{t_{on}+t_{off}}$, where *t_on_* is the time that the valve remains open and *t_off_* is the time the valve remains closed. To demonstrate the pump action, the *t_on_* values of each of the 3 valves for a pump device were fixed at 1 s, while the *t_off_* values were varied from 1 to 5 s to achieve different frequencies.

## 3. Results

### 3.1. Baseline Flow Rate Characterization in a Heat-Bonded PMMA-FWF Straight Channel Device

To establish a reference for the performance of more complex microfluidic devices, we characterized the simplest device configuration: a straight channel. This experiment was designed to evaluate the flow rate as a function of input flow pressure (1–20 psi) in a device assembled by heat bonding PMMA slabs with a food-wrap film (FWF) membrane sandwiched between the layers (see Figure 5D). The results from this baseline experiment provide key performance metrics that can be compared with future microfluidic designs involving more complex geometries and functional components.

**Figure 6:**
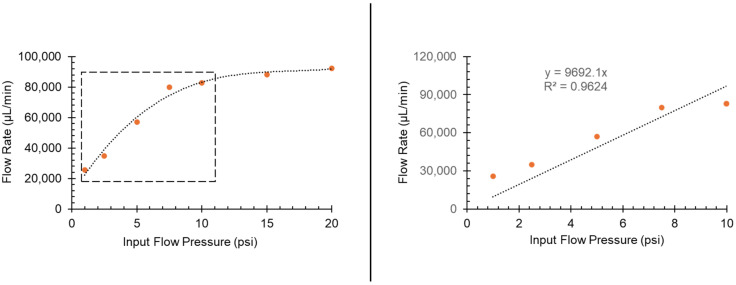
Flow rate as a function of input flow pressure for a baseline straight channel microfluidic device prepared by heat bonding PMMA and FWF layers. (**Left**) The full range of the input flow pressure- dashed square box shows the linear region. (**Right**) The zoomed region indicating the 0–10 psi part of the plot. All flow rates are calculated by measuring the volume of the liquid collected at the device’s outlet and dividing it by the time that it took to collect the volume.

Specifically, Figure 6 illustrates the relationship between input pressure and the resulting flow rate to assess the straight channel’s performance under varying pressure conditions. The flow rate was determined using the volumetric displacement of fluid over time across different input pressures. As shown, the flow rate increases almost linearly between 1 and 10 psi, rising from approximately 2500 µL/min to 83,000 µL/min. After the 10 psi mark, the flow rate plateaued at around 92,000 µL/min. This trend is characteristic of straight channels, where flow resistance decreases with increasing pressure but eventually levels off at higher pressures. This plateau suggests that the channel approaches its maximum capacity for fluid transport under the given pressure conditions. These results establish key operational parameters for the straight channel microfluidic device and assembled by heat bonding PMMA slabs with the FWF membrane and serve as a performance benchmark for more complex designs. Additionally, the calculated Reynolds numbers for the device used in Figure 6 were estimated to be Re ~838 at 1 psi and Re ~3024 at 20 psi. These values indicate that within the tested range, the device operates predominantly under laminar flow, with transitional behavior emerging at higher flow rates, which is critical for some applications such as optimizing analyte transport and sensor response. A more detailed calculation with a table summarizing the results is shown in the Supplementary file (see Supplementary Table S1).

### 3.2. Determination of Closing Pressure for the FWF Valve via Leakage Characterization

**Figure 7:**
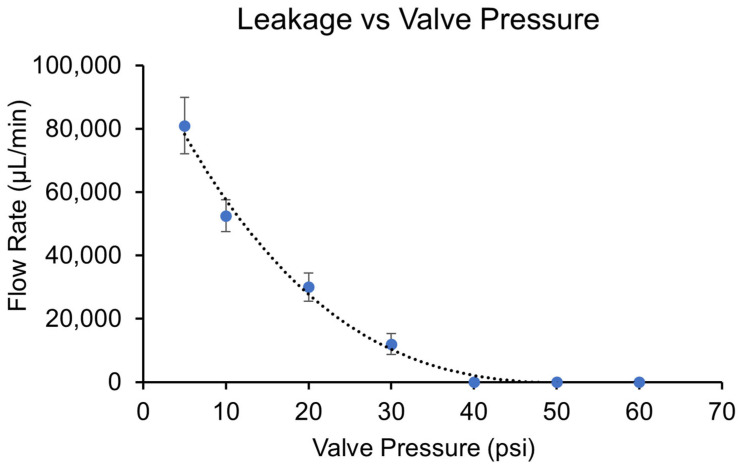
The leakage flow rate as a function of FWF valve control pressure. The graph depicts how the leakage flow rate decreases with increasing input pressure applied to the FWF valve’s control channel. The flow rate drops sharply between 0 and 40 psi, indicating improved valve sealing, and approaches zero around 50 psi, demonstrating the effective closure of the valve. Data points represent mean values; error bars show ± standard deviation (*n* = 5).

To evaluate the effectiveness of the valve seal in our microfluidic device, we measure the leakage as a function of input pressure applied to the valve’s control channel. As the air pressure is increased in the control channel, we expect the valve to create a tighter seal, reducing the leakage flow rate until complete closure is achieved. This test is critical in determining the pressure threshold required for effective valve operation, as well as understanding any limitations in sealing performance. By plotting the flow rate at the device outlet against the input pressure, we can visualize the leakage behavior across a range of pressures (see Figure 7).

As shown in Figure 7, the leakage flow rate decreases exponentially as the input pressure increases. Between 0 and 40 psi, the flow rate drops significantly, falling from 81,000 µL/min at 0 psi to about 12,000 µL/min at 30 psi. And beyond 40 psi, the leakage flow rate approaches zero, indicating that the valve has effectively sealed. These results suggest that the valve requires a minimum of around 40 psi to achieve full closure and prevent leakage, providing an operational benchmark for pressure-driven valve control in this system. While these results demonstrate effective valve closure, direct comparison with TPU performance under identical conditions was not possible due to the material procurement limitations that motivated this study.

For comparison, this required pressure is significantly higher than the ~30 psi needed to fully close a similar valve made out of PDMS—the gold standard material for microfluidic device fabrication [20]. A likely explanation is that PVDC, the primary component of the FWF, has a Young modulus that is 3–6 times higher than that of the PDMS, specifically in the range of 3.45–3.79 GPa for PVDC, compared to 1.32 to 2.97 MPa for those of PDMS [21].

This marked difference in mechanical properties likely explains why a higher pressure is needed to achieve complete valve closure when using the FWF membrane. Nevertheless, the FWF’s sealing mechanism under high pressure is robust, achieving a tight and impermeable seal. The optimization of the device’s channel dimensions and laser engraving process further ensures reliable valve function, which is crucial for precise fluid control in future applications.

### 3.3. Durability and Burst Pressure of the FWF-Based Microvalves

The durability and reliability of microvalves in microfluidic systems are critical factors in determining their practical utility. In this study, we performed extensive testing to evaluate the performance of our microvalves under repeated cycling conditions. Our results indicate that after approximately 1,000,000 cycles over a period of 21 days, the microvalves showed no signs of leakage. This was a similar level of durability to what was reported for the TPU membrane [14,22,23] and two orders of magnitude higher than what was reported for PDMS valves. These results confirm the stability and compatibility of the FWF membrane for repeated use under the testing conditions. Throughout the cycling process, with an input pressure of 50 psi maintained consistently, no deterioration in the membrane’s performance was observed. The fatigue testing was conducted at 0.5 Hz, which was representative of biological applications but may not have captured high-frequency viscoelastic behavior relevant to industrial microfluidic systems.

Additionally, burst pressure testing was conducted to determine the maximum pressure that the FWF valves could withstand. Our PMMA-FWF microfluidic devices were burst-tested by adding pressure to the valves and channels up to 60 psi, which corresponded to the highest pressure supplied by our laboratory’s house air. Even at this maximum pressure, no signs of leakage or device failure were observed. 

Compared to previously reported membrane materials and bonding strategies, this performance was highly competitive. For instance, the study by S. Bhattacharya’s group showed that PDMS–PDMS bonding via high-density Inductively Coupled Plasma (ICP) and Plasma-Enhanced Chemical Vapor Deposition (PECVD) supports up to 58 psi, while PDMS–glass or silicon bonds can reach 74 psi under similar surface activation conditions [24]. PDMS–PMMA interfaces show more variation depending on the treatment method used: chemical glue with plasma yields > 72.5 psi, while advanced coating and compression bonding can exceed 90 psi, according to Zhang et al. [25]. In contrast, other PDMS–thermoplastic combinations and bonding strategies relying on room-temperature silane chemistry yield lower strengths (e.g., 50–65 psi for PDMS–PC or PDMS–PET-Polyethylene Terephthalate) [26]. 

In bonding configurations more analogous to membrane applications, polyurethane-based films show more modest results, typically in the range of 6–47 psi depending on bonding strategy [e.g., plasma, dip-coating, or microcontact printing [27]. While direct comparison is complicated by differences in device geometry, bonding method, and testing protocols, our results demonstrate that PMMA-FWF microfluidic device assembly via thermal fusion bonding is a viable alternative to conventional materials like PDMS, glass, PMMA, TPU, PU, PC, PET, etc., and their combinations, offering competitive burst resistance while supporting reliable, leak-free operation in a compact, membrane-integrated format. This can make PMMA-FWF suitable for practical applications and large-scale production.

### 3.4. Application of FWF Membrane Valves: On-Chip Peristaltic Pumping System

In addition to its use as a membrane for Quake-style microvalves, the FWF can be utilized in micropumps, employing a peristaltic mechanism to pump fluids without the need for external pumps. In this setup, three valves of identical size were placed next to each other and programmed to open and close in sequence, thereby pushing the fluid through the channel (see Video S1 in Supporting Information).

**Figure 8:**
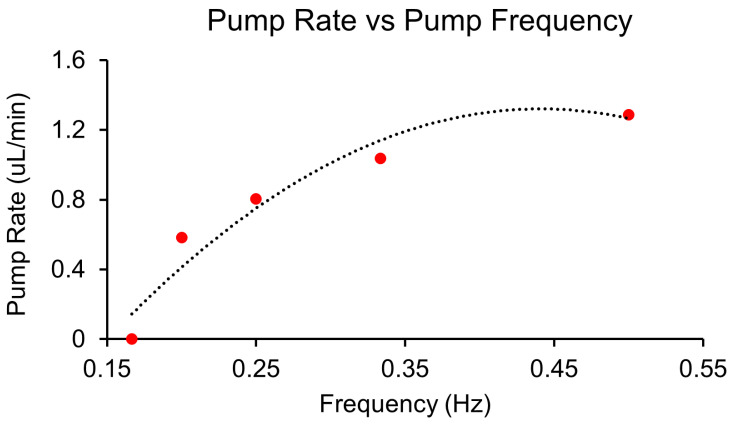
The pump rate as a function of pumping frequency for a peristaltic microfluidic pump. The graph shows the pump rate (µL/min) achieved by the FWF-based peristaltic pump system at varying operational solenoid frequencies (Hz). The three-valve pump system demonstrated a maximum flow rate of 1.33 µL/min at 1 Hz, with a declining pump rate noted as the frequency increased. The performance compared well with that of a similar peristaltic pump system using commercial thermoplastic polyurethane (TPU), demonstrating the viability of FWF as a cost-effective alternative for on-chip pumping applications.

The pump performance of the FWF-based peristaltic microfluidic system was evaluated across a range of operational frequencies. As shown in Figure 8, the pump rate increased as the pumping frequency increased, with the maximum flow rate of 1.33 µL/min achieved at the fastest frequency (of the current solenoid valve) of 0.5 Hz and valve pressure of 40 psi. This expected pump behavior could be attributed to the wave generation of the mechanism of the peristaltic pump, as faster frequencies corresponded to faster waves, which led to a greater volume of fluid displacement, thus leading to a higher pump rate.

When compared to a peristaltic pump system using a commercial TPU membrane, the FWF-based pump demonstrated comparable performance, achieving similar flow rates under equivalent operating conditions. This finding underscores the feasibility of using the FWF membrane as a low-cost (an estimation of the cost of FWFs in comparison with other membrane-application materials is provided in Supplementary Table S2) alternative to TPU while maintaining the required operational standards for microfluidic applications. The system also maintained consistent performance without failure within the 1,000,000-cycle expected lifetime of the FWF membrane.

These functional demonstrations establish FWF-based peristaltic pumps as suitable for real-world applications including automated reagent delivery in point-of-care diagnostics, sample preparation in lab-on-chip systems, and continuous perfusion in cell culture platforms.

## 4. Conclusions

This study demonstrates the viability of food-wrap film (FWF) as a cost-effective and readily available alternative to thermoplastic polyurethane (TPU) membranes in microfluidic devices, specifically for on-chip valves and pumps. At an approximate cost of USD 1/m^2^, FWF significantly reduces material expenses compared to specialized laboratory polymers like TPU, offering researchers an accessible and easily integrable solution. The adoption of FWF thus represents a potential breakthrough in democratizing access to microfluidic technology, lowering entry barriers, particularly in resource-limited settings, for rapid prototyping, and for educational purposes.

FWF membranes exhibited reliable sealing capabilities with valve closure pressures around 40 psi, stable peristaltic pump flow rates of up to approximately 1.33 μL/min, and robust mechanical durability, withstanding over 1,000,000 actuation cycles without performance deterioration. Additionally, burst pressure testing confirmed that FWF valves could tolerate pressures exceeding 60 psi without leakage, demonstrating the robustness of the thermal bonding method used for device assembly. These metrics qualitatively align with published TPU data, although direct side-by-side comparison was not feasible due to TPU procurement limitations. Future studies with direct access to TPU materials would benefit from quantitative comparisons to further validate performance equivalence.

The thermal resistance of FWF, tolerating temperatures up to 140 °C, facilitates integration into PMMA-based devices through reliable thermal bonding without risk of deformation. The straightforward fabrication process underscores FWF’s suitability for large-scale microfluidic device production. The demonstrated peristaltic pumping capability addresses practical fluid handling needs in diagnostic devices, analytical systems, and cell culture applications, highlighting FWF’s broad utility.

Despite these advantages, FWF presents limitations. It requires relatively large channel dimensions (approximately 0.940 mm width and 0.4 mm depth), as the material does not flex sufficiently for smaller microfluidic channels. Additionally, the relatively high closing pressure of around 40 psi limits FWF’s suitability for low-pressure microfluidic systems, such as some point-of-care devices. Nonetheless, this closing pressure remains within the typical range for laboratory compressed air supplies (~60 psi).

Future research should investigate FWF performance under high-frequency actuation (>100 Hz) to characterize its frequency-dependent viscoelastic properties and expand potential application domains. Moreover, comprehensive studies on chemical compatibility and biocompatibility with diverse reagents and biological systems are needed to fully elucidate the operational scope of FWF.

This study addresses a critical gap in the literature by providing the first alternative material with similar properties to TPU for laser-cut microfluidic membrane applications, filling a previously unmet need due to TPU’s procurement challenges. Future work will focus on integration testing within specific application workflows to demonstrate complete system performance in practical scenarios, further optimizing design and operational parameters to enhance FWF-based microfluidic technology.

## Data Availability

The original contributions presented in this study are included in the article/Supplementary Material. Further inquiries can be directed to the corresponding authors.

## Supplementary Materials

The following supporting information can be downloaded at https://www.mdpi.com/article/10.3390/mi16060657/s1; Figure S1: Representative COMSOL simulation result showing complete membrane deformation for fully occluded channel case. Von Mises stress distribution (color scale) overlaid on deformed geometry demonstrates parabolic deflection profile with maximum displacement at channel center under 40 psi applied pressure; Figure S2: Channel closure percentage as a function of channel width. COMSOL simulation results showing increasing membrane deflection (negative Y-direction displacement) with larger channel dimensions under 40 psi applied pressure. Negative percentages indicate downward membrane displacement relative to initial horizontal position; Figure S3: Membrane deflection profiles across relative channel width for different channel geometries (0.7–1.1 mm). Curves show spatial distribution of membrane deformation with maximum deflection occurring at channel center (50% relative width). Negative percentages represent downward displacement in negative Y-direction under 40 psi applied pressure; Figure S4: FWF-PMMA microfluidics device layout. (A) Actual layers of pre-assembled de-vice, in corresponding to (B) CAD design of the 2 layers and scale bar, and (C) Zoom-in CAD design of the air bubble trap; Table S1: Reynolds numbers table for the device used in Figure 6; Table S2: Estimated cost comparison of common materials used as membrane for microvalves and micro-pumps of microfluidics devices. Estimated cost is based on general search, excluding factors like unique properties (permeability, thickness, custom mechanical properties, etc.); Video S1: FWF membrane-based peristaltic micropump demonstration.

## Abbreviations

The following abbreviations are used in this manuscript:

FWF	Food-Wrap Film
TPU	Thermoplastic Polyurethane
PMMA	Polymethyl Methacrylate
IPA	Isopropyl Alcohol
PVDC	Polyvinylidene Chloride
CO2	Carbon Dioxide
NJIT	New Jersey Institute of Technology
PTFE	Polytetrafluoroethylene
PDMS	Poly(dimethylsiloxane)
ICP	Inductively Coupled Plasma
PECVD	Plasma-Enhanced Chemical Vapor Deposition
PC	Polycarbonate
PET	Polyethylene Terephthalate
PS	Polystyrene
COC	Cyclic Olefin Copolymer
Q	Flow Rate
V	Volume
t	Time
ton/toff	Valve On/Off Time
NIH	National Institutes of Health
NSF	National Science Foundation
