# Supplementary material for: Common Food-Wrap Film as a Cost-Effective and Readily Available Alternative to Thermoplastic Polyurethane (TPU) Membranes for Microfluidic On-Chip Valves and Pumps
Source: Micromachines (Basel). 2025 May 30;16(6):657. doi: 10.3390/mi16060657 (PMC12195204; doi:10.3390/mi16060657)
Supplement: Supplementary file 1 [file micromachines-16-00657-s001.zip › Supplementary_File_Micromachines_MDPI_Food_wrap.pdf]

SUPPLEMENTARY INFORMATION

I. COMSOL Finite Element Analysis of FWF Membrane Deformation

1.1. Model Description

A two-dimensional finite element model was developed in COMSOL Multiphysics 6.2 to simulate the mechanical deformation of food wrap film (FWF) membranes under applied pressure. The model geometry consisted of a rectangular membrane domain with dimensions corresponding to the channel width (0.7-1.1 mm parametric range) and membrane thickness (16 µm). Material properties were assigned based on experimental characterization of FWF: Young's modulus of 3.55 GPa, Poisson's ratio of 0.35, and density of 1400 kg/m^3.

Boundary conditions included fixed constraints at the membrane edges (representing bonding to the rigid substrate) and a follower pressure load of 40 psi applied to the top surface. The solid mechanics physics module solved for displacement and stress fields using a stationary solver with automatic mesh refinement. A parametric sweep was performed across channel widths from 0.7 to 1.1 mm in 0.05 mm increments to characterize the geometric dependence of membrane deformation.

1.2. Results and Analysis

The simulation successfully modeled complete channel occlusion under applied pressure loading. Figure S1 demonstrates a representative case of full membrane deformation in a 0.95mm wide channel, showing the characteristic parabolic deflection profile with maximum displacement at the channel center and gradual transition to fixed boundary conditions at the edges. The stress distribution visualization confirms efficient load transfer through the membrane structure.

Figure S2 shows the relationship between channel width and overall channel closure percentage, demonstrating increasing closure (more negative values) with larger channel widths. Figure S3 presents deflection profiles across the relative channel width for each geometric configuration, showing the spatial distribution of membrane deformation.

The negative percentages in Figures S2 and S3 represent downward displacement of the membrane in the negative Y-direction relative to its initial horizontal position. In Figure S2, measurements represent maximum deflection along the entire channel width. In Figure S3, measurements are taken at the channel center where maximum deflection occurs, revealing the characteristic parabolic deformation pattern expected for pressure-loaded membranes with fixed edge constraints.

The quantitative results establish clear design criteria for FWF valve operation, with closure percentages ranging from -40% to -190% across the tested geometric range. This finite element analysis validates the controllable, predictable nature of FWF membrane deformation and provides essential engineering parameters for optimizing microfluidic valve performance.

II. Reynolds Number Calculation for Device in Figure 6 of Main Manuscript

Formulations used for the Reynolds number calculation:

$$Re = \frac{\rho \,.\, v \,.\, D_h}{\mu}$$

In which, ρ = fluid density (kg/m³), v = average fluid velocity (m/s), D_h = hydraulic diameter of the channel (m), μ = dynamic viscosity of the fluid (Pa·s)

With water-diluted fluid dye, we assume that the fluid is water at 20ºC, with density **ρ: 997 kg/m³**, dynamic **μ** viscosity: **0.89×10−3 Pa·s**

Average **fluid velocity** is calculated from flow rate as follows:

$$\boldsymbol{v} = \frac{Q}{A}$$

In which, v = average fluid velocity (m/s), Q = fluid flow rate (μL/min- converted to m^3/s), and A = cross-sectional area (m^2).

The cross-sectional area of the channel is illustrated as follows:

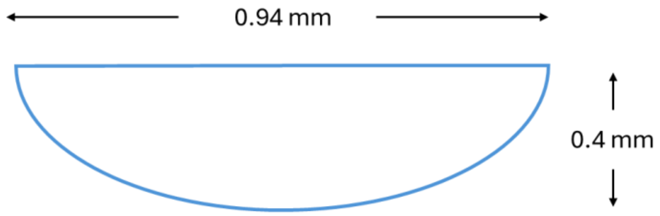

Which is a arc-segment round channel, not a perfect semi-circular channel. We assume the area in the formula:

$$A = \frac{R^2}{2}.(\theta - sin\theta)$$

In which A = cross-sectional area (m^2). The two values of R = arc radius (m), and θ (rad) is calculated as:

$$R = \frac{h}{2} + \frac{w^2}{8h}$$

In which h is the height of the channel at deepest point (0.4 mm), w is the width of the channel (0.94 mm). This will result in the R = 0.000476 m

$$\theta = 2.cos^{-1}\left(\frac{R-h}{R}\right)$$

This will result in $\theta = 2.821$ rad

Together, A is calculated to be 2.845×10^{-7} (m^2)

The wetted perimeter P is calculated as follows:

$$P = R.\theta + w$$

This will result in P = 0.002283 (m)

To calculate the hydraulic diameter of the arc-segment channel, we follows the formula:

$$D_h = \frac{4.A}{P}$$

This will result in **D_h = 0.000498 (m)**

III. SUPPLEMENTARY FIGURES

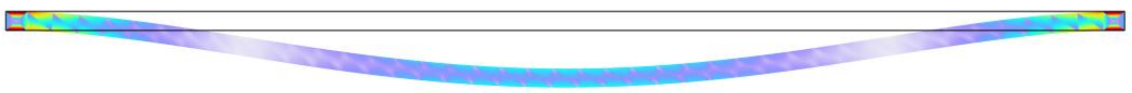

Figure S1. Representative COMSOL simulation result showing complete membrane deformation for fully occluded channel case. Von Mises stress distribution (color scale) overlaid on deformed geometry demonstrates parabolic deflection profile with maximum displacement at channel center under 40 psi applied pressure.

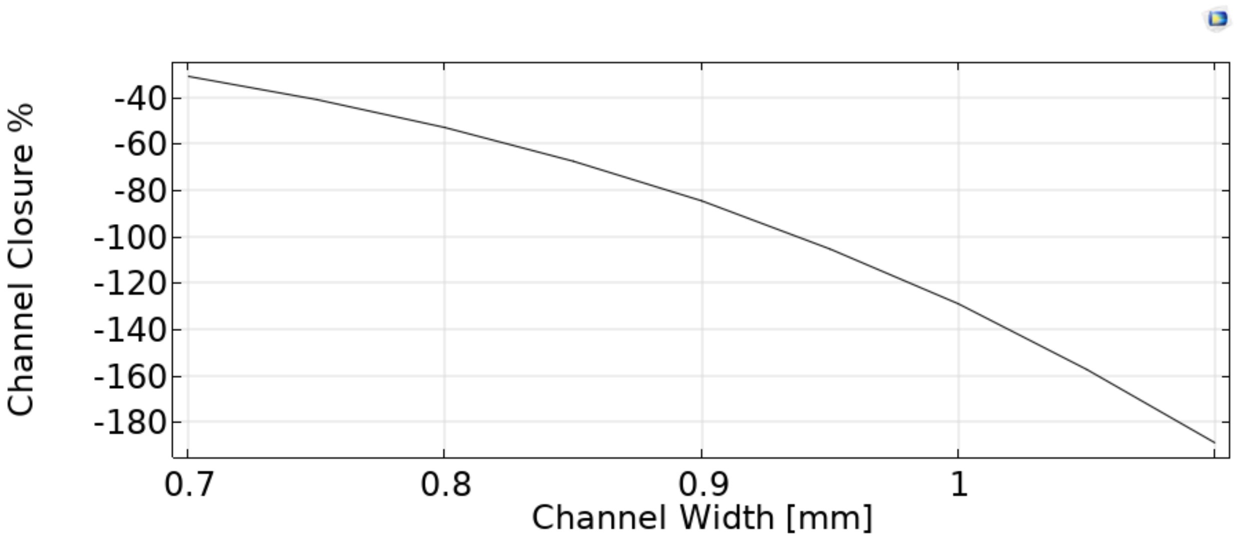

Figure S2. Channel closure percentage as a function of channel width. COMSOL simulation results showing increasing membrane deflection (negative Y-direction displacement) with larger channel dimensions under 40 psi applied pressure. Negative percentages indicate downward membrane displacement relative to initial horizontal position.

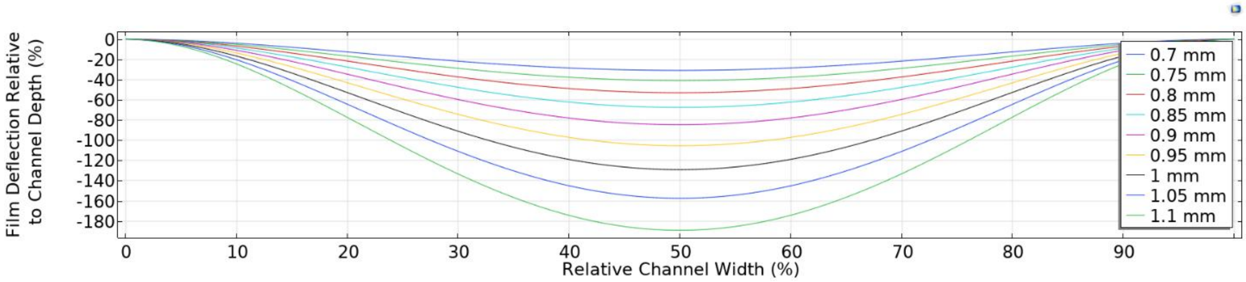

Figure S3. Membrane deflection profiles across relative channel width for different channel geometries (0.7-1.1 mm). Curves show spatial distribution of membrane deformation with maximum deflection occurring at channel center (50% relative width). Negative percentages represent downward displacement in negative Y-direction under 40 psi applied pressure.

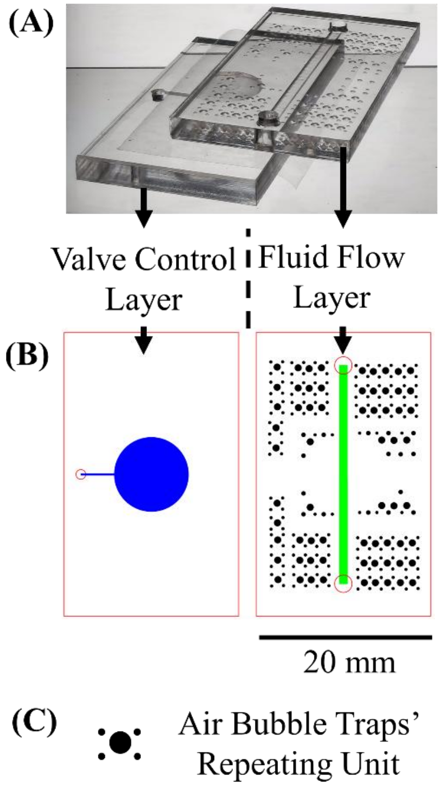

Figure S4. FWF-PMMA microfluidics device layout. (A) Actual layers of pre-assembled device, in corresponding to (B) CAD design of the 2 layers and scale bar, and (C) Zoom-in CAD design of the air bubble trap.

IV. SUPPLEMENTARY TABLES

Table S1. Reynolds numbers table for the device used in Figure 6.

Pressure (psi)	Q (µL/min)	Re
20	92,445	3,024
15	88,102	2,881
10	82,908	2,712
7.5	79,881	2,613
5	57,157	1,869
2.5	34,943	1,143
1	25,622	838

Table S2. Estimated cost comparison of common materials used as membrane for microvalves and micropumps of microfluidics devices. Estimated cost is based on general search, excluding factors like unique properties (permeability, thickness, custom mechanical properties, etc.):

Membrane Material	Approximate Cost per m²	Notes
**FWF (Food Wrap Film)**	~$1	Commercial-grade polyethylene or PVC; widely available, lower mechanical/chemical robustness.
**Medical-grade PDMS**	~$1,500–$3,000	Typically custom-fabricated (spin-coated); high purity, biocompatibility, and flexibility; not sold in bulk sheet form.
**Medical-grade TPU**	~$200–$800	High-purity TPU membranes (thickness ~50–150 µm); used in implantables and drug-delivery systems; e.g., Lubrizol, Covestro medical TPU films.
**Silicone Rubber Sheets**	~$100–$250	Commercially available thin sheets (e.g., 0.1–0.5 mm); flexible and elastic; used as substitutes for PDMS in prototyping.
